# The *Non-Coding RNA* Journal Club: Highlights on Recent Papers-8

**DOI:** 10.3390/ncrna7010023

**Published:** 2021-03-19

**Authors:** Sonia Tarallo, Barbara Pardini, Archa H. Fox, Hayley Ingram, Cristian Taccioli, Joseph H. Taube, Sendurai A. Mani

**Affiliations:** 1 Italian Institute for Genomic Medicine (IIGM), c/o IRCCS Candiolo, 10060 Candiolo, Turin, Italy; sonia.tarallo@iigm.it; 2 Candiolo Cancer Institute—FPO IRCCS, 10060 Candiolo, Turin, Italy; 3 School of Human Sciences, The University of Western Australia, 35 Stirling Highway, Crawley, WA 6009, Australia; 22346861@student.uwa.edu.au; 4 School of Molecular Sciences, The University of Western Australia, 35 Stirling Highway, Crawley, WA 6009, Australia; 5 Department of Animal Medicine, Health and Production, University of Padova, 35133 Padova, Provincia di Padova, Italy; 6 Department of Biology, Baylor University, Waco, TX 76798, USA; Joseph_Taube@baylor.edu; 7 Department of Translational Molecular Pathology, MD Anderson Cancer Center, Houston, TX 77030, USA

## 1. Introduction

We are glad to share with you our eighth Journal Club and to highlight some of the most interesting papers published recently. We hope to help you keep aware of non-coding RNA research outside of your area. The *Non-Coding RNA* Scientific Board wishes you an exciting and fruitful read.

## 2. Charting Extracellular Transcriptomes in The Human Biofluid RNA Atlas

### Highlight by Sonia Tarallo and Barbara Pardini

In recent years, researchers have started to literally wander in an enormous, mostly unknown, universe of different RNA species (mostly non-coding) in biofluids as potential biomarkers for diseases. However, a clear image of the RNA representation and abundance in different specimens is still missing, and no criteria for validation and reproducibility have been assessed. The situation is even more complex since there is no consensus regarding the collection of biofluids, the way non-coding RNAs are transported in the matrix (freely circulating or in extracellular vesicles), the techniques used for transcriptome assessment and the pipeline of analyses, as well as the best site/specimen to be investigated according to the disease.

Researchers have tried to create some order in this complex universe. The recent work of Hulstaert et al. [1] reported their effort in building an atlas of different RNA species measured in 20 different human biofluids with an innovative sequencing technique that measured not only circular RNAs (circRNAs) and microRNAs but also messenger RNAs (mRNAs), which are usually hard to measure in biofluids. Interestingly, the authors introduced innovative methods of spike-in controls to precisely compare RNA content across biofluids. This approach revealed an amazing 10,000-fold difference in concentration among specimens and the use of spike-ins resulted in a data normalization that better reflected the biological situation in the presence of a disease neutralizing the global differences in abundance across biofluids. The authors reported that each transcriptome was enriched in a variegated series of RNA species derived from specific tissues and cell types, with circRNAs being more abundant in biofluids than linear forms.

As a final goal, the data could be a reference for the selection of the most relevant biofluids to monitor particular diseases and also give some guidelines for better analyses and comparisons among biomarker studies in extracellular matrices. 

## 3. Live Cell RNA Imaging by CRISPR–Cas13 Systems

### Highlight by Archa H. Fox and Hayley Ingram 

Despite the success of RNA detection techniques in fixed-cell experiments, the conventional ways of tracking RNA in live cells are limited, being non-specific and complicated to establish. A 2019 publication by Ling-Ling Chen’s group in *Molecular Cell* has provided a breakthrough, demonstrating an advance in live cell non-coding RNA imaging utilizing CRISPR–Cas13 systems [2]. 

MS2-MCP live cell RNA detection has limitations with ease of use, RNA structure issues and/or expression in widespread applications. Rectifying this, the authors described the use of dPspCas13b and dPguCas13b for robust imaging of prominent coding and non-coding RNA transcripts including NEAT1 (Nuclear Paraspeckle Assembly Transcript 1), MUC4 (Mucin 4), GCN4 (General Control Non-depressible 4) and SatIII (Satellite III repeated sequences) in live cells. Using this new method, with GFP-dPspCas13b and guide RNAs targeting NEAT1, the authors rapidly and efficiently observed NEAT1-labeled paraspeckle dynamics, revealing a consistency with a “kiss-and-run/fusion” model after de novo formation. Combinatorial experiments also demonstrated viable single-cell, dual-color RNA imaging and simultaneous RNA/DNA visualization by combining dCas13 with MS2-MCP or dCas9 systems, respectively. 

As the localization and dynamics of non-coding RNAs are critical for their function, this improved visualization method is important for advancing the field. Further experiments may aim to improve the capability of this technique through additional Cas13 protein identification. 

## 4. Involvement of Non-Coding RNAs and Transcription Factors in the Induction of Transglutaminase Isoforms by ATRA

### Highlight by Cristian Taccioli

Type 2 transglutaminase (TGM2) is a ubiquitous enzyme associated with neurodegenerative inflammatory pathologies, celiac disease and cancer progression. Furthermore, it is also related to epithelial–mesenchymal transition, supporting motility, metastasis, survival and drug resistance. 

The enzyme structure and catalytic activities of TGM2 have been investigated for many years, and recently, Franzese et al. found that two long non-coding RNAs (lncRNAs) transcribed within this gene are able to regulate the expression of its isoforms that are related to the presence of specific transcription factors particularly in ATRA-exposed leukemic (NB4, HL-60) and neuronal (SK-N-SH, SH-SY5Y) cell lines [3]. 

In particular, the GATA3 emerged as the driver of the expression of one of these lncRNAs located within TGM2 gene. Moreover, through the binding to phUPF1, a component of NMD responsible for the active degradation of targets, this lncRNA was found to protect other transcripts from degradation. 

In addition, based on RNA immunoprecipitation experiments, Franzese et al. found that this lncRNA interacts with RNApol II, RXRα and Max, both regulatory factors of TGM2. Finally, Franzese et al. concluded that TGM2 lncRNAs are engaged in the generation/accumulation of altered variants with dramatically different catalytic activities, especially detected in pathological conditions.

## 5. *MaTAR lncRNA* Binds Purine-Rich DNA and Impacts Extracellular Matrix to Drive Breast Cancer Progression

### Highlight by Joseph. H. Taube and Sendurai A. Mani

Numerous long non-coding RNAs (lncRNAs) implicated in breast cancer progression and metastasis have been well characterized, including HOX antisense intergenic RNA (HOTAIR), Metastasis Associated Lung Adenocarcinoma Transcript 1 (MALAT1), Phosphatase 1 Nuclear-Targeting Subunit (PNUTS) and NF-κB-interacting long noncoding RNA (NKILA). Excitingly, locked nucleic acid (LNA) technology is beginning to facilitate the translation of these findings into potential interventions. Ground-breaking new work from David Spector’s group at Cold Spring Harbor Labs illustrates a new key player in breast cancer progression: *MaTAR25* (mouse)/*LINC01271* (human). Like many other lncRNAs, *MaTAR25* is enriched in the nucleus and interacts with chromatin; however, Chang et al. [4] identified a complex of proteins that interact with purine-rich regions as a novel means of genomic targeting. *MaTAR25* and its protein binding partners (PURA, PURB and YBX1) are recruited to many genomic loci, one of which is *TNS1*, which encodes for the Tensin protein, a mediator of cell-to-matrix adhesion and migration. Genetic loss-of-function analysis using *MaTAR25* knockout 4T1 breast cancer cells demonstrates its necessity for adhesion-associated cellular phenotypes, including migration and invasion in vitro and primary tumor growth and metastasis in vivo. Demonstrating the translational advancements in this field, Chang et al. proceeded to administer anti-sense oligos to knock down *MaTAR25* and demonstrate suppressed tumor growth. This pathway is highly relevant to humans as the ortholog *LINC01271* is more highly expressed in advanced stages of breast cancer and is highly expressed in lung metastases. 
